# Target attainment of cefotaxime in critically ill children with meningococcal septic shock as a model for cefotaxime dosing in severe pediatric sepsis

**DOI:** 10.1007/s10096-019-03535-w

**Published:** 2019-04-09

**Authors:** Stan J. F. Hartman, Navin P. Boeddha, Ebru Ekinci, Birgit C. P. Koch, Rogier Donders, Jan A. Hazelzet, Gertjan J. Driessen, Saskia N. de Wildt

**Affiliations:** 1 Department of Pharmacology-Toxicology, Radboudumc, Geert Grooteplein Zuid 10, 6525 GA Nijmegen, The Netherlands; 2 Intensive Care and Department of Pediatric Surgery, Erasmus MC-Sophia Children’s Hospital, University Medical Center Rotterdam, Rotterdam, The Netherlands; 3 Department of Pediatrics, Division of Pediatric Infectious Diseases and Immunology, Erasmus MC-Sophia Children’s Hospital, University Medical Center Rotterdam, Rotterdam, The Netherlands; 4 Department of Pharmacy, ErasmusMC, Rotterdam, The Netherlands; 5 Department for Health Evidence, Radboudumc, Nijmegen, The Netherlands; 6 Department of Pediatrics, Juliana Children’s Hospital, Haga Teaching Hospital, The Hague, The Netherlands

**Keywords:** Cefotaxime, Pharmacokinetics, Target attainment, Critically ill children, Sepsis

## Abstract

Reduced target attainment of β-lactam antibiotics is reported in critically ill patients. However, as target attainment of cefotaxime in severely ill pediatric sepsis patients may differ from adults due to age-related variation in pharmacokinetics, we aimed to assess target attainment of cefotaxime in this pilot study using meningococcal septic shock patients as a model for severe sepsis. Secondary analysis of prospectively collected data from a randomized controlled trial. Children with meningococcal septic shock (1 month to 18 years) included in this study received cefotaxime 100–150 mg/kg/day as antibiotic treatment. Left-over plasma samples were analyzed using LC-MS/MS to determine cefotaxime concentrations. MIC values from EUCAST were used to determine target attainment of cefotaxime for Neisseria meningitidis (0.125 mg/l), but also for *Streptococcus pneumoniae* (0.5 mg/l), Enterobacteriaceae (1 mg/l), and *Staphylococcus aureus* (4 mg/l). Target attainment was adequate when all samples exceeded MIC or fourfold MIC values. One thirty-six plasma samples of 37 severe septic shock patients were analyzed for cefotaxime concentrations. Median age was 2 years with a median PRISM-score of 24 and mortality of 24.8%. The median unbound cefotaxime concentration was 4.8 mg/l (range 0–48.7). Target attainment ranged from 94.6% for the MIC of N. meningitidis to 16.2% for fourfold the MIC *S. aureus*. Creatinine levels were significantly correlated with cefotaxime levels. Target attainment of cefotaxime with current dosing guidelines seems to be adequate for N. meningitidis but seems to fail for more frequently encountered pathogens in severely ill children.

## Introduction

Studies on the target attainment of β-lactam antibiotics in critically ill adult patients indicate that up to 41% do not achieve adequate plasma levels [1]. Pharmacokinetic studies in critically ill children have shown reduced target attainment of several, frequently used, antibiotics as well [2–4]. To our knowledge, only one recent study investigated target attainment of cefotaxime, in a relatively stable PICU cohort [5].

This reduced target attainment is a direct result of pathophysiological changes during critical illness, causing an increase in the volume of distribution, clearance, or both [6]. The risk of non-target attainment is particularly high for β-lactam antibiotics, due to their time-dependent kill characteristics [7].

Meningococcal sepsis is characterized by a severe, rapid onset of sepsis and multi-organ failure, with a high mortality rate (25–30%) and long-term sequelae in 11–19% of survivors [8]. Although the incidence of meningococcal sepsis has rapidly declined due to vaccination for *Neisseria meningitidis*, data from these patients can serve as a model for severe sepsis in critically ill children and guide optimal antibiotic dosing.

The aim of this study was to identify target attainment of cefotaxime for *N. meningitidis* in critically ill children with meningococcal sepsis and to extrapolate to target attainment for other frequently encountered pathogens in pediatric sepsis.

## Materials and methods

### Setting

We conducted a secondary analysis of data from a randomized, double-blinded, placebo-controlled phase 2 trial (RCT) designed to assess the activation process of protein C in critically ill children with meningococcal septic shock [9]. Children were randomized to receive placebo or protein C in addition to standard care for septic shock including antibiotic treatment with cefotaxime 100–150 mg/kg/day in 3–4 doses.

Thirty-eight children aged 1 month to 18 years were recruited in the PICU of the Erasmus MC- Sophia Children’s Hospital, Rotterdam, The Netherlands. The inclusion and exclusion criteria were detailed in the original publication [9]. The RCT had been approved by the Erasmus MC medical ethics review board and all parents or legal representatives had signed informed consent for the use of left-over materials in follow-up research.

Patient characteristics and clinical parameters had been prospectively collected in the context of the RCT, including age, weight, gender, disease severity scores (SOFA-score and PRISM-score), and mortality. Data on antibiotic dosing orders and co-medication were collected from the hospital records.

Blood samples were taken at PICU admission, 6, 12, and 24 h after admission and once daily thereafter. After analysis for the RCT, plasma samples were stored at − 80 °C for secondary use.

### Drug analysis

Cefotaxime concentrations in plasma were quantified with a UPLC–MS/MS system consisting of a Dionex Ultimate UPLC system connected to a triple quadrupole mass spectrometer (Thermo TSQ Vantage with HESI-probe, Thermo Scientific) [10]. The lower and upper levels of quantification of this method were 0.13 and 12.5 mg/l, respectively.

### Target attainment

β-lactam antibiotics, including cephalosporins like cefotaxime, show a time-dependent pathogenic kill potential, with a pharmacodynamic target of fT>MIC > 60–70%, meaning that concentrations should be above the MIC for at least 60–70% of the time [11]. However, recent literature proposes fT>MIC targets of 100%, associated with better clinical outcome in critically ill patients [12] and even fT>4xMIC for a maximum killing potential in critically ill and immunocompromised patients [13].

Antibiotic plasma concentrations were compared with the clinical breakpoint for susceptibility of *Neisseria meningitidis* to cefotaxime (0.125 mg/l) from the European Committee of Antimicrobial Susceptibility Testing (EUCAST) [14]. Furthermore, to extrapolate our findings to other infections, comparisons were made to clinical breakpoints of *Streptococcus pneumoniae* (0.5 mg/l), *Enterobacteriaceae* (1.0 mg/l), and *Staphylococcus aureus* (4 mg/l), the most common causative pathogens of pediatric sepsis [15].

Since the RCT was not designed for cefotaxime PK analysis, samples were regarded to be randomly scavenged across the cefotaxime dosing interval. Furthermore, since only total drug concentrations were determined, we assumed 60% of the total concentration to be the unbound fraction of cefotaxime [16].

Two approaches to define target attainment were used as outcome measures: firstly, unbound plasma concentrations were compared with MIC and fourfold MIC values of the studied pathogens to define the percentage of samples that exceeded these target MICs. Secondly, we identified the percentage of patients at risk as patients with any unbound cefotaxime concentrations below the MIC or fourfold MIC during their ICU stay.

### Statistical analysis

Data were analyzed with IBM SPSS Statistics for Windows, Version 22.0. Demographic data were analyzed using descriptive statistics and presented as “median (IQR, range)” for continuous variables and “absolute number (percentage)” for dichotomous variables.

Linear regression was performed to identify relevant covariates of cefotaxime concentrations (age, PRISM-score, SOFA-score, and serum creatinine levels). In the cases of multiple values for a single patient, i.e., for creatinine levels, data were analyzed using generalized estimating equations (GEE) to correct for within-patient variation and multiple sampling. In the case of missing data, values were imputed by linear extrapolation from adjacent data points.

## Results

### Patient characteristics

A total of 136 plasma samples of 37 out of 38 patients in the original RCT were available for cefotaxime analysis (1–7 samples per patient). Patients had a median age of 2 years (range 0.3–16.1 years) and a median weight of 13.7 kg (range 6–70 kg). Median PRISM scores were 24 (range 4–43) and the mortality rate during admission was 24.3% (Table 1).

**Table 1:** Demographic information of patients

Demographic variables	All patients (*n* = 37)	
Age (years)	2.0 years (IQR 0.9–8.3, range 0.3–16.1)	
Age groups	0–2 years:	18 (48.6%)
	6–12 years:	7 (18.4%)
	2–6 years:	10 (26.3%)
	12–18 years:	2 (5.3%)
Sex	M 21 (56.8%)	
	F 16 (43.2%)	
Weight (kg)	13.7 kg (IQR 10–30, range 6–70)	
PRISM-score	24 (IQR 17–30, range 4–43)	
SOFA-score	10 (IQR 8–14, range 4–19)	
Mortality *n* (%)	9/37 (24.3%)	
Duration of ICU admission (days)^*a*^	4.0 days (IQR 2.3–7.0, range 1–45)	
Cefotaxime dose (mg/kg/day)	150 mg/kg/day (IQR 150–160, range 133–600)	
Receiving more than 1 antibiotic agent (cefotaxime + rifampicin)	4/37 (10.8%)	
Positive culture for *Neisseria meningitidis*	32/37 (84.2%)	
Creatinine levels (μmol/l) ^*b*^	41 μmol/l (IQR 24.3–73.8, range 7–462)	

^a^: analysis done on the surviving patients (*n* = 28),

^b^: creatinine levels determined by Jaffé method

*PRISM* predicted risk of mortality score, *SOFA* sequential organ failure assessment score

Continuous variables are presented as median (IQR, range)

Dichotomous variables are presented as *n* (%)

### Drug levels and target attainment

Total cefotaxime plasma concentration varied widely, with a median of 8.0 mg/l (IQR 2.5–18.7 mg/l and range 0–81.1 mg/l). When accounting for protein binding, median unbound plasma concentrations were 4.8 mg/l (range 0–48.7 mg/l). Cefotaxime concentrations were below the lower level of quantification in six samples, five of which had been taken at PICU admission (*t* = 0), in patients having received another cephalosporin.

**Fig. 1:**
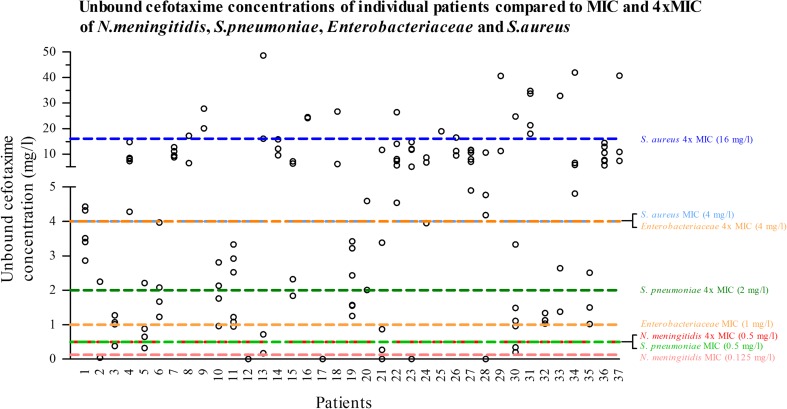
Plasma concentrations of cefotaxime for each individual patient with reference lines for the MIC and fourfold MIC of *N. meningitidis* (red), *S. pneumoniae* (green)*, Enterobacteriaceae* (orange), and *S.aureus* (blue). The amount of samples and the number of patients with one or more samples above the MIC or fourfold MIC can be found in Tables 2 and 3, respectively

The proportion of samples above the MIC of *N. meningitides* was 95.6% and above the MIC of *S. pneumoniae, Enterobacteriaceae,* and *S. aureus* in 91.2%, 86.0%, and 55.1% of samples, respectively. Using the higher target (fT>4xMIC), unbound plasma concentrations were above fourfold MIC of *N. meningitidis* in 91.2% of samples and of *S. pneumoniae, Enterobacteriaceae,* and *S. aureus* in 71.3%, 55.1%, and 14.7%, respectively (Table 2 and Fig. 1).

**Table 2:** Percentage of samples with unbound concentrations above the MIC and fourfold MIC of selected pathogens

Analysis	*N. meningitidis*		*S. pneumoniae*		*Enterobacteriaceae*		*S. aureus*	
	MIC (0.125 mg/l)	4xMIC (0.5 mg/l)	MIC (0.5 mg/l)	4xMIC (2 mg/l)	MIC (1 mg/l)	4xMIC (4 mg/l)	MIC (4 mg/l)	4xMIC (16 mg/l)
*n* (%) of samples above target concentration	130/136 (95.6%)	124/136 (91.2%)	124/136 (91.2%)	97/136 (71.3%)	117/136 (86.0%)	75/136 (55.1%)	75/136 (55.1%)	20/136 (14.7%)

The proportion of patients with all of their samples above the MIC for *N. meningitides*, *S. pneumoniae*, *Enterobacteriaceae*, and *S. aureus* was 94.6%, 86.5%, 75.7%, and 51.4%, respectively. For the higher target of fT>4xMIC, this ranged from 86.5% for *N. meningitides* to 16.2% for *S. aureus* (Table 3).

**Table 3:** Percentage of patients with all unbound plasma concentrations above the MIC and fourfold MIC of selected pathogens

Analysis	*N. meningitidis*		*S. pneumoniae*		*Enterobacteriaceae*		*S. aureus*	
	MIC (0.125 mg/l)	4xMIC (0.5 mg/l)	MIC (0.5 mg/l)	4xMIC (2 mg/l)	MIC (1 mg/l)	4xMIC (4 mg/l)	MIC (4 mg/l)	4xMIC (16 mg/l)
*n* (%) of patients with all samples above target concentration	35/37 (94.6%)	32/37 (86.5%)	32/37 (86.5%)	22/37 (59.5%)	28/37 (75.7%)	19/37 (51.4%)	19/37 (51.4%)	6/37 (16.2%)

### Co-variate analysis

**Fig. 2:**
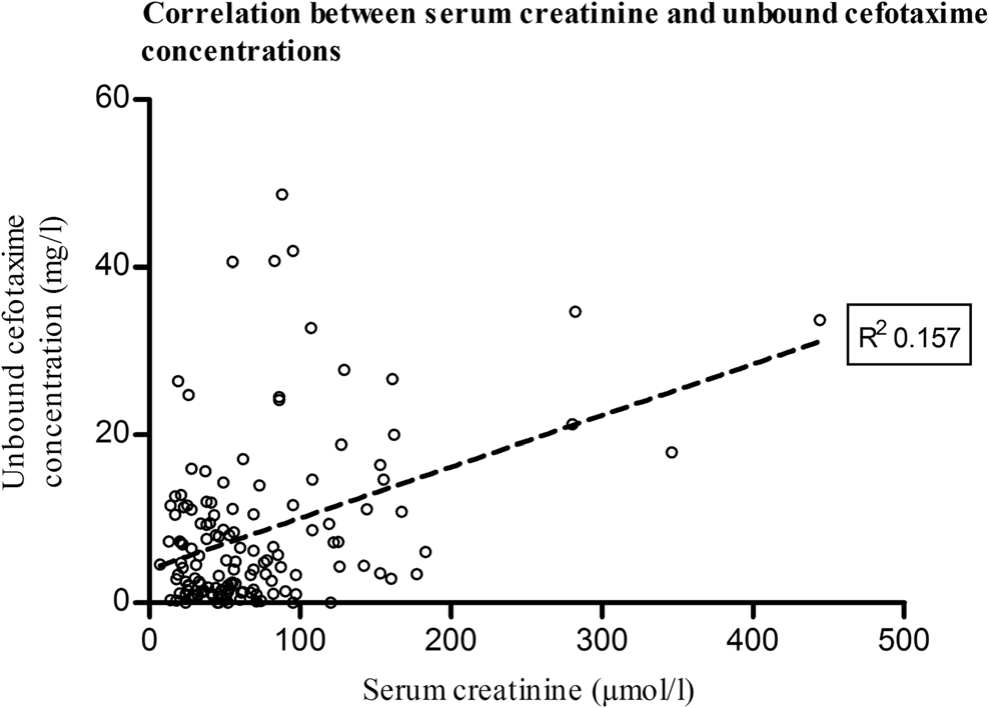
Correlation between serum creatinine and unbound plasma concentrations of cefotaxime. Legend: each dot represents a single cefotaxime concentration measurement with accessory serum creatinine concentration. The solid black line represents the linear regression line (*p* < 0.001, *R*^2^ = 0.157)

The relationships of age, PRISM-score, SOFA-score, and creatinine levels with cefotaxime plasma levels were explored using linear regression. Of these studied variables, only creatinine levels were weakly but significantly correlated with cefotaxime levels (*R*^2^ = 0.157, *p* < 0.001), as shown in Fig. 2. This association was maintained when correcting for repeated measurements within subjects using GEE (*B* = 0.061 (95% CI 0.041–0.081, *p* < 0.001).

## Discussion

In this secondary analysis of prospectively collected data, we evaluated the hypothesis that pathophysiological changes in critically ill children with sepsis could lead to low target attainment of cefotaxime. To our knowledge, this is the first cohort of severe pediatric septic shock patients in which cefotaxime concentrations are reported.

Although most samples were well above the MIC of *N. meningitidis*, even when a higher target of four times the MIC is pursued, target attainment for less susceptible pathogens was poor, even with the most conservative target of fT>MIC of 100%. Therefore, using cefotaxime in current doses as blind, broad-spectrum antibiotic therapy might lead to therapy failure in children with severe sepsis caused by less susceptible pathogens.

These results are in line with recent population pharmacokinetic studies that show that critically ill children require a higher dose of β-lactam antibiotics compared with non-critically ill patients [2–7]. The study by Béranger et al. concludes that the target of fT>MIC 100% is not reached with standard intermittent cefotaxime dosing in critically ill children [5]. An important difference with this population pharmacokinetic study is that our patients were more severely ill as is reflected by much higher mortality in our cohort (24.3% vs. 2.0%). Furthermore, kidney function was not included in their model while in our study creatinine correlated significantly, albeit weakly, with cefotaxime concentrations. This can be explained by a greater variation in kidney function in our cohort (range of creatinine 7–472 μmol/l in our cohort vs. 11–81 μmol/l). However, the large inter-individual variation in cefotaxime concentrations cannot be contributed to clearance alone, but could be explained by additional changes in the volume of distribution.

Béranger and colleagues propose continuous dosing to reach target attainment for patients of any age and weight. Although continuous infusion is possible on the PICU, it remains cumbersome in routine care. Moreover, meta-analyses of RCTs in adults are inconclusive whether continuous infusion of β-lactam antibiotics leads to a survival benefit over intermittent dosing [17, 18].

Our findings suggest that antibiotic dosing guidelines should incorporate pharmacodynamic endpoints, e.g., MIC values. Current standard doses result in median unbound plasma concentrations 40-fold above the targeted values for highly susceptible pathogens like *N. meningitidis*, but for less susceptible pathogens even the most conservative target is rarely reached. Incorporating appropriate pharmacodynamic endpoints in dosing guidelines can offer tailored doses for both susceptible and less susceptible pathogens. A recent study by Woksepp et al. investigated target attainment of β-lactam antibiotics in critically ill adults based on the MIC of the cultured pathogen, compared with EUCAST clinical breakpoints [19]. They found markedly higher target attainment when using the true-MIC (89%) compared with clinical breakpoints (55%), which also highlights that data on susceptibility could provide valuable information to improve antibiotic dosing regimens.

Our study shows some limitations: Firstly, samples were randomly scavenged and were not collected with the purpose of evaluating drug disposition. However, since the pharmacodynamic target for β-lactam antibiotics is to maintain drug concentrations above the MIC for the entire dosing interval, our results represent a “best-case scenario” of target attainment of cefotaxime for these pathogens in critically ill children with severe septic shock.

Secondly, samples have been stored for a prolonged period (maximum 19 years) at – 80 °C and underwent a single freeze-thaw cycle that could impact sample quality. A study comparing the effect of storage temperature for 11 different β-lactam antibiotics, including cefotaxime, shows that cefotaxime was stable after 52 weeks at – 70 °C [20]. In addition, the concentrations in our study were similar to those reported in critically ill adults [21]. Thirdly, we did not determine concentrations of cefotaxime’s active metabolite desacetyl-cefotaxime, which does contribute to the antibiotic effect, but we believe it has limited influence on overall cefotaxime exposure and consequent effect [22].

## Conclusion

In this secondary analysis of a prospective RCT, we have analyzed the target attainment of cefotaxime in critically ill children. Target attainment of cefotaxime seems adequate for susceptible pathogens but poor for less susceptible pathogens. Therefore, using cefotaxime with the current dosing guidelines as blind, broad-spectrum antibiotic therapy might lead to therapy failure. These findings suggest a benefit of incorporating pharmacodynamic endpoints in dosing guidelines for antibiotics. Prospective research is needed for an adequate analysis of target attainment of cefotaxime in critically ill children and identification of relevant covariates to design individualized dosing guidelines.
